# Safety and efficacy of day-case hip and knee arthroplasty in the NHS: a nationwide UK cohort study

**DOI:** 10.1186/s42836-026-00377-8

**Published:** 2026-03-13

**Authors:** Omar Musbahi, Ahmed Al-Saadawi, Saran Singh Gill, Sara Sousi, Alex Bottle, Justin P. Cobb, Gareth G. Jones

**Affiliations:** 1 MSk Lab, Sir Michael Uren Hub, White City Campus, Imperial College London, London, W12 0BZ UK; 2 Lister Hospital, East and North Hertfordshire Teaching NHS Trust, Coreys Mill Ln, Stevenage, SG1 4AB UK; 3 School of Public Health, White City Campus, Imperial College London, London, W12 0BZ UK

**Keywords:** Day case surgery, Ambulatory, Arthroplasty, Safety

## Abstract

**Introduction:**

In recent years, day-case hip and knee arthroplasty has emerged as a potential solution to the elective backlog within the NHS. While international literature on this topic is extensive, only a handful of single-centre studies have been conducted in the United Kingdom. This study aimed to examine the safety and efficacy of day-case hip and knee arthroplasty in the UK using a 20-year linked national NHS dataset.

**Methods:**

A cohort study was conducted using the Clinical Practice Research Datalink (CPRD), linked to Hospital Episode Statistics (HES) and the Office for National Statistics death registry. Adults undergoing primary hip or knee arthroplasty between 1998 and 2021 were included. Procedures were classified as day-case or inpatient, using two distinct approaches: patient classification and length of stay. Day-case is defined as discharge on the same day of the procedure, while inpatient procedures involve undergo at least one overnight stay in hospital. The primary outcomes assessed were A&E attendance, readmission, and critical care admission within 90-days post-operatively. Secondary outcomes included 90-day complication rates and survival analysis. Propensity score matching was implemented to adjust outcomes for age, gender, comorbidity burden, deprivation index, and ethnicity.

**Results:**

In total, 1,822 (0.16%) procedures were classified as day-case, while 4,355 (0.37%) had a recorded length of stay of 0 days. On average, patients undergoing day-case arthroplasty were younger, more often male, and had fewer comorbidities than their inpatient counterparts. Higher rates of A&E attendance (12% vs 9.1%; *P* = 0.001) and readmission (5.7% vs 3.7%; *P* < 0.001) were observed in the day-case cohort. In contrast, deep vein thrombosis (0.5% vs 0.9%; *P* = 0.010) and infection rates (1.0% vs 1.9%; *P* = 0.014) were lower in this patient group. Survival analyses demonstrated significantly higher adjusted survival probabilities associated with day-case arthroplasty (HR: 0.84; [95% CI: 0.72–0.99]; *P* = 0.034) over a 20-year follow-up period.

**Conclusion:**

Day-case hip and knee arthroplasty has been demonstrated to be  safe and feasible, with comparable complication rates to the traditional inpatient setting. However, within the context of the NHS, it is currently associated with higher rates of 90-day A&E attendance and readmission. While increasing day-case volumes may help address elective backlogs, it is important to ensure that the appropriate patient selection criteria, optimised peri-operative care, and post-discharge support are in place before this approach is expanded in the UK.

**Supplementary Information:**

The online version contains supplementary material available at 10.1186/s42836-026-00377-8.

## Introduction

Hip and knee arthroplasty are among the most commonly performed elective procedures worldwide, with over 100,000 of each undertaken annually in the United Kingdom [1]. With an ageing population, this figure is expected to continue rising in the coming years [2]. Additionally, the COVID-19 (Coronavirus Disease 2019) pandemic resulted in an unprecedented reduction in total hip (THA) and knee arthroplasty (TKA) between 2019 and 2021 [3]. Consequently, the average waiting times for these procedures, from referral, nearly doubled in 2021 and have yet to return to pre-pandemic levels [4]. This leaves many patients with worsening pain, functional impairment, and a reduced quality of life [5]. In the 2025 *Reforming Elective Care* plan, NHS England highlighted day-case hip and knee arthroplasty as a key component of its strategy to address the elective backlog, and its consequences [6]. Day-case is defined as discharge on the same day of the procedure, while inpatient procedures involve undergo at least one overnight stay in the hospital [7]. Enabling a shift in practice towards day surgery would be especially beneficial within the NHS, as the UK has one of the lowest hospital bed numbers per capita in Europe, with approximately 19 of every 20 beds occupied at all times [8].

Approximately 0.55% of THAs, 0.52% of TKAs, and 5.44% of UKAs are performed as day-case procedures within the NHS, figures considerably lower than those reported in the United States (USA) [9]. There is significant evidence suggesting that day-case arthroplasty offers safe and comparable outcomes to the traditional inpatient model, while being more efficient and cost-effective [10–12]. Additionally, it is associated with high levels of patient satisfaction, which is particularly helpful in the current timeline of declining patient experience due to the aforementioned challenges [13].

Nonetheless, concerns remain regarding the safety of this approach, given that the majority of evidence so far is from relatively small cohort studies, with heterogeneous data, and an inherent selection bias in the day-case groups towards younger, lower BMI patients with minimal comorbidities [14]. This is particularly the case with data from the UK, which is limited to single-centre studies with short-term follow-up [15–17]. The present study sought to address these limitations by using a linked NHS dataset covering the entire United Kingdom and encompassing over a million patients with long-term follow-up. This study aimed to examine the safety and efficacy of day-case hip and knee arthroplasty in the UK, comparing various clinical outcomes, including post-operative complications, readmission, A&E attendance, and survival.

## Methods

### Study design and data source

We conducted a retrospective cohort study using data obtained from the Clinical Practice Research Datalink (CPRD), linked to Hospital Episode Statistics (HES) and the Office for National Statistics (ONS) death registry, which provided detailed data on causes and dates of death between 2nd January 1998 and 29th March 2021. Inclusion criteria were adult patients undergoing TKA, UKA, THA, and hip resurfacing arthroplasty (HRA) between 1998 and 2021, who were identified using OPCS-4 procedure codes. Patient were excluded based on the following criteria: (1) missing gender, (2) age < 18, (3) missing IMD data, (4) invalid death data (i.e., death date prior to the procedure), (5) those with missing operation dates, (6) simultaneous hip and knee procedures, (7) inconsistencies in episode date coding (i.e., start date after end date), (8) inconsistencies in admission date coding (i.e., admission date after discharge date or episode start date), (9) discharge date prior to episode end date, (10) non valid operation dates (i.e., outside 1998–2021), and (11) operations without a patient classification code. Procedures were categorised by hospital coders as day-case or non-day-case using the “CLASSPAT” code based on the patient classification (PC) flag, which reflects the planned mode of management. Additionally, to capture patients who were treated as a day-case, but might have been assigned an incorrect PC code, we also used the hospital length of stay (LoS), defined as the number of days from admission to discharge, to identify patients with zero-day LoS (considered a day-case procedure) versus those with non-zero LoS (non-day-case).

### Statistical analyses

Baseline characteristics for day-case and non-day-case patients were summarised as counts and percentages for categorical variables, and medians with interquartile ranges (IQR) for continuous variables. Group differences were assessed using Pearson’s Chi-squared test for categorical variables and the Wilcoxon rank sum test for continuous variables. Covariates included age at operation, gender, ethnicity, geographical region, Index of Multiple Deprivation (IMD 2019), and modified Elixhauser Comorbidity Index (ECI) over the 5 years prior to surgery using HES [18, 19], body mass index (BMI), and type of arthroplasty.

### Primary outcomes

The primary post-operative outcomes included 90-day A&E attendance, readmission, and critical care admission based on HES critical care data. Bivariate analyses were also performed to compare 90-day outcomes between groups.

### Secondary outcomes

Secondary post-operative outcomes included pulmonary embolism (PE), deep vein thrombosis (DVT), and all-cause infection (including sepsis), as defined using ICD-10 diagnostic codes (Table S1). Specifically, a diagnosis of DVT was confirmed by a proximal leg vein ultrasound. Bivariate analyses were also performed to compare 90-day outcomes between groups.

A survival analysis was also conducted and analysed as a secondary outcome. Kaplan–Meier (KM) survival curves were generated to visualise time to death, stratified by day-case status (day-case vs. non-day-case) and LoS (zero vs. non-zero days). Log-rank tests and 95% confidence intervals (CIs) were reported. Univariable and multivariable Cox proportional hazards models were used to estimate hazard ratios (HRs) for overall survival, adjusting for age, gender, IMD, ECI score, and surgery type. All analyses were conducted using R (version 4.4.1). This study and CPRD data access were approved by the Medicines and Healthcare products Regulatory Agency Independent Scientific Advisory Committee (reference ID: 22_002170).

Additionally, variation in day-case procedures for lower limb joint arthroplasty across consultants was analysed as a secondary outcome. For each consultant, the total number of operations, the number of day-cases, and the proportion of procedures performed as day-cases were calculated. A chi-squared test was used to evaluate differences in the distribution of day versus non-day-case procedures across regions.

### Propensity score matching

Propensity score matching was performed using nearest neighbour matching with a 4:1 ratio (inpatient: day-case) to balance potential cofounders, including age, gender, ethnicity, ECI, surgery type, and IMD. Propensity scores were estimated using logistic regression. Covariate balance before and after matching was assessed using standardised mean differences (SMD), with SMD < 0.1 considered acceptable balance. Matching was performed for both the coded procedure groups and the LoS groups. The incidence of 90-day outcomes was compared between the two groups using Pearson’s Chi-squared test, and Fisher’s exact test was applied where expected cell counts were below five.

## Results

In total, 1,182,211 procedures were identified, of which 1,148,293 met the inclusion criteria and were included in the final analysis. Patients were excluded based on the following criteria: (1) missing gender (*n* = 15), (2) age < 18 (*n* = 4,516), (3) missing IMD data (*n* = 14,050), (4) invalid death data (*n* = 198), (5) missing operation dates (*n* = 0), (6) simultaneous hip and knee procedures (*n* = 287), (7) inconsistencies in episode date coding (*n* = 27), (8) inconsistencies in admission date coding, i.e., admission date after discharge date (*n* = 1,008) or after episode start date (*n* = 198), (9) discharge date prior to episode end date (*n* = 322), (10) non-valid operation dates (*n* = 13,292), and (11) operations without a patient classification code (*n* = 5). Of these, 1,822 (0.16%) were coded as day-case procedures, and 1,820 (99.9%) of these also had a recorded LoS of 0 days. In total, there were 4,355 (0.38%) procedures recorded as having a LoS of zero days. A statistically significant difference in the proportion of arthroplasty procedures performed as day cases was observed in both the *patient classification* and *LoS* datasets, with UKA having the highest proportion of day-case procedures in both (Table 1).

**Table 1:** Proportion of day-case procedures stratified by arthroplasty type

	**HRA**	**THA**	**TKA**	**UKA**	***P*-value**^***2***^
	**N = 14,156**^***1***^	**N = 538,730**^***1***^	**N = 540,284**^***1***^	**N = 55,123**^***1***^	
**Patient Classification**					** < 0.001**
Inpatient	14,124 (99.8%)	538,237 (99.9%)	539,736 (99.9%)	54,374 (98.6%)	
Day-case	32 (0.2%)	493 (0.1%)	548 (0.1%)	749 (1.4%)	
**LoS**					** < 0.001**
> 0	14,105 (99.6%)	537,339 (99.7%)	538,853 (99.7%)	53,641 (97.3%)	
=0	51 (0.4%)	1,391 (0.3%)	1,431 (0.3%)	1,482 (2.7%)	

^*1*^n (%); ^*2*^Pearson’s Chi-squared test

### Patient demographics

The mean age of patients undergoing day-case arthroplasty was 64 years, significantly lower than the average of 70 years in the inpatient cohort (*P* < 0.001). Additionally, a significantly higher proportion of patients in the day-case cohort were male compared to their counterparts (*P* < 0.001). Regarding socioeconomic factors, both geographical region and IMD status differed significantly between day-case and inpatient groups (*P* < 0.001). A significant difference in pre-operative ECI score was also observed between the two cohorts, with a higher proportion of day-case patients having the lowest score, as well as a lower proportion with the highest score (*P* < 0.001) (Table 2).

**Table 2:** Patient demographics

**Covariate**	**Inpatient Pathway**	**Day-Case Pathway**	***P*-value**^***2,3***^	**LoS > 0**	**LoS = 0**	***P*-value**^*2*^
	**N = 1,146,471**^***1***^	**N = 1,822**^***1***^		**N = 1,143,938**^***1***^	**N = 4,355**^***1***^	
**Age at Operation (years)**	70 (62, 77)	64 (54, 72)	< 0.001***	70 (62, 77)	65 (56, 73)	< 0.001***
**Gender**			< 0.001***			< 0.001***
Female	691,654 (60%)	970 (53%)		690,341 (60%)	2,283 (52%)	
Male	454,817 (40%)	852 (47%)		453,597 (40%)	2,072 (48%)	
**Ethnicity**						
Black	13,160 (1.1%)	25 (1.4%)		13,128 (1.1%)	57 (1.3%)	
Chinese	596 (< 0.1%)	2 (0.1%)		592 (< 0.1%)	6 (0.1%)	
Mixed	2,832 (0.2%)	1 (< 0.1%)		2,830 (0.2%)	3 (< 0.1%)	
Other	10,530 (0.9%)	6 (0.3%)		10,497 (0.9%)	39 (0.9%)	
South Asian	20,718 (1.8%)	22 (1.2%)		20,676 (1.8%)	64 (1.5%)	
Unknown	189,534 (17%)	635 (35%)		189,058 (17%)	1,111 (26%)	
White	909,101 (79%)	1,131 (62%)		907,157 (79%)	3,075 (71%)	
**Region**			< 0.001***			< 0.001***
East Midlands	27,874 (2.4%)	116 (6.4%)		27,828 (2.4%)	162 (3.7%)	
East of England	52,977 (4.6%)	44 (2.4%)		52,899 (4.6%)	122 (2.8%)	
London	146,039 (13%)	196 (11%)		145,644 (13%)	591 (14%)	
North East	38,971 (3.4%)	66 (3.6%)		38,806 (3.4%)	231 (5.3%)	
North West	199,385 (17%)	274 (15%)		199,002 (17%)	657 (15%)	
South East	264,959 (23%)	508 (28%)		264,126 (23%)	1,341 (31%)	
South West	165,824 (14%)	249 (14%)		165,557 (14%)	516 (12%)	
West Midlands	205,876 (18%)	172 (9.4%)		205,601 (18%)	447 (10%)	
Yorkshire and the Humber	44,566 (3.9%)	197 (11%)		44,475 (3.9%)	288 (6.6%)	
**IMD 2019**			< 0.001***			< 0.001***
1 (Most deprived)	128,755 (11%)	215 (12%)		128,429 (11%)	541 (12%)	
2	125,647 (11%)	235 (13%)		125,336 (11%)	546 (13%)	
3	130,486 (11%)	235 (13%)		130,178 (11%)	543 (12%)	
4	133,805 (12%)	232 (13%)		133,505 (12%)	532 (12%)	
5	116,897 (10%)	190 (10%)		116,650 (10%)	437 (10%)	
6	121,131 (11%)	181 (9.9%)		120,912 (11%)	400 (9.2%)	
7	113,029 (9.9%)	135 (7.4%)		112,792 (9.9%)	372 (8.5%)	
8	96,537 (8.4%)	141 (7.7%)		96,341 (8.4%)	337 (7.7%)	
9	94,454 (8.2%)	129 (7.1%)		94,254 (8.2%)	329 (7.6%)	
10 (Least deprived)	85,730 (7.5%)	129 (7.1%)		85,541 (7.5%)	318 (7.3%)	
**Modified ECI**			< 0.001***			< 0.001***
< 0	73,485 (6.4%)	137 (7.5%)		916,675 (80%)	3,614 (83%)	
0	780,191 (68%)	1,286 (71%)		163,161 (14%)	565 (13%)	
1–5	176,716 (15%)	287 (16%)		40,723 (3.6%)	124 (2.8%)	
6–13	88,000 (7.7%)	91 (5.0%)		23,379 (2.0%)	52 (1.2%)	
14 +	28,079 (2.4%)	21 (1.2%)		28,041 (2.5%)	59 (1.4%)	
**BMI**	28.3 (25.1, 32.1)	28.3 (25.2, 32.1)	0.867	28.3 (25.1, 32.1)	28.4 (25.3, 32.4)	0.258
Unknown	668,506 (58.3%)	1,071 (58.8%)		667,001 (58.3%)	2,576 (59.2%)	
**Surgery Type**			< 0.001***			< 0.001***
HRA	14,124 (1.2%)	32 (1.8%)		14,105 (1.2%)	51 (1.2%)	
THA	538,237 (47%)	493 (27%)		537,339 (47%)	1,391 (32%)	
TKA	539,736 (47%)	548 (30%)		538,853 (47%)	1,431 (33%)	
UKA	54,374 (4.7%)	749 (41%)		53,641 (4.7%)	1,482 (34%)	

^*1*^n (%); ^*2*^Pearson’s Chi-squared test; ^*3*^Fisher’s exact test; *P < 0.05; **P < 0.01; ***P < 0.001

Similarly, in the LoS dataset, patients with a LoS of 0 days were, on average, younger, more likely to be male, more deprived according to IMD status, and had more favourable pre-operative ECI scores compared to those with longer hospital stays (*P* < 0.001). The remaining baseline patient demographics, including stratification by surgery type, can be seen in Table 2.

### Trends in day-case arthroplasty

**Fig. 1:**
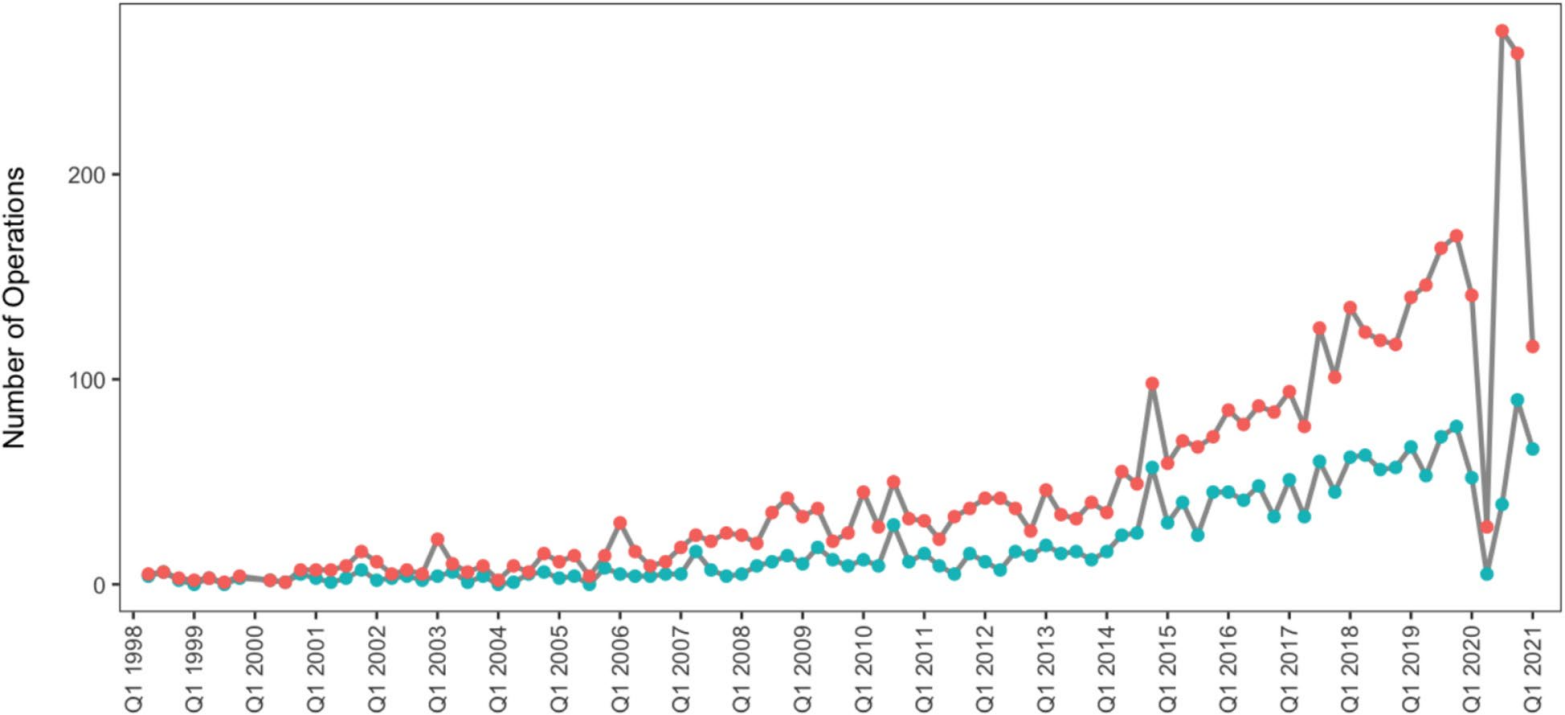
Temporal trends in day-case hip and knee arthroplasty rates. *Red: Inpatient procedures; Teal: Day-case procedures*

Figure 1 illustrates a steady increase in the volume of day-case hip and knee arthroplasty. The upward trajectory was met by a sharp decline in 2020, corresponding with the onset of the COVID-19 pandemic. This was followed by an even greater rise, reaching an all-time high in the second half of 2020.

**Fig. 2:**
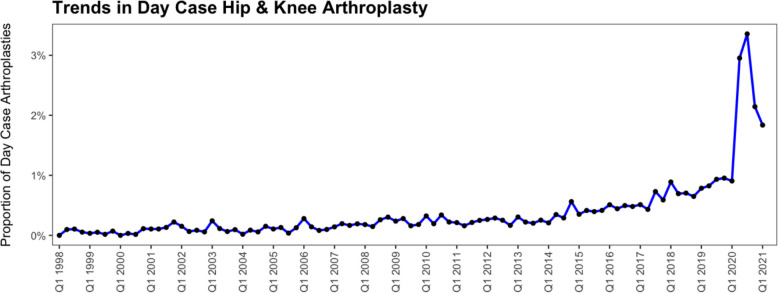
Temporal trends in the proportion of hip and knee arthroplasty performed as day-case procedures

Similarly, the ratio of day-case to non-day-case procedures has steadily risen over the years, with a marked rise in 2020, most likely attributable to the COVID-19 pandemic (Fig. 2).

### 90-day post-operative clinical outcomes

As seen in Table 3, for patients classified as planned day-case procedures, A&E attendance (*P* = 0.016) and readmission rates (*P* = 0.004) were higher among the day-case cohort. However, rates of infection (*P* < 0.001) were statistically significantly lower among day-case patients compared to their inpatient counterparts.

**Table 3:** Comparison of 90-day clinical outcomes between day-case and inpatient procedures

90-day Outcomes	Inpatient Pathway	Day-Case Pathway	*P*-value^*2,3*^	LoS > 0	LoS = 0	*P*-value^*2*^
	**N = 1,146,471**^***1***^	**N = 1,822**^***1***^		**N = 1,143,938**^***1***^	**N = 4,355**^***1***^	
**Any AE attendance**	112,196 (9.8%)	209 (11%)	0.016*	111,905 (9.8%)	500 (11%)	< 0.001***
**AE attendance resulting in admission**	51,494 (4.5%)	107 (5.9%)	0.004**	51,317 (4.5%)	284 (6.5%)	< 0.001***
**Critical Care**	32,890 (2.9%)	60 (3.3%)	0.278	32,794 (2.9%)	156 (3.6%)	0.005**
**PE**	7,457 (0.7%)	7 (0.4%)	0.158	7,445 (0.7%)	19 (0.4%)	0.079
**DVT**	9,970 (0.9%)	11 (0.6%)	0.222	9,959 (0.9%)	22 (0.5%)	0.010**
**Infection**	30,167 (2.6%)	19 (1.0%)	< 0.001***	30,121 (2.6%)	65 (1.5%)	< 0.001***

^*1*^n (%); ^*2*^Pearson’s Chi-squared test; ^*3*^Fisher’s exact test; *P < 0.05; **P < 0.01; ***P < 0.001

Within the LoS dataset, similar patterns were observed, with higher rates of A&E attendance (*P* < 0.001) and readmission (*P* < 0.001). However, in addition, critical care admission was also higher among patients discharged on the same day (*P* = 0.005). In this dataset, deep vein thrombosis (*P* = 0.010) and infection rates (*P* < 0.001) were significantly lower among those with shorter stays (Table 3).

### Propensity score-matched 90-day clinical outcomes

After propensity score matching, A&E attendance (*P* = 0.001) and readmission rates (*P* < 0.001) remained higher within the day-case cohort, while infection rates were lower (*P* = 0.014) (Table 4**)**. All the aforementioned statistically significant associations were replicated in the LoS dataset (A&E attendance and readmission: *P* < 0.001; infection: *P* = 0.011).

**Table 4:** Propensity score-matched comparison of 90-day clinical outcomes between day-case and inpatient procedures

90-day Outcomes	Inpatient Pathway	Day-Case Pathway	*P*-value^*2,3*^	LoS > 0	LoS = 0	*P*-value^*2,3*^
	**N = 7,288**^***1***^	**N = 1,822**^***1***^		**N = 17,420**^***1***^	**N = 4,355**^***1***^	
**Any AE attendance**	694 (9.5%)	209 (11%)	0.013*	1,689 (9.7%)	500 (11%)	< 0.001***
**AE attendance resulting in admission**	249 (3.4%)	107 (5.9%)	< 0.001***	651 (3.7%)	284 (6.5%)	< 0.001***
**Critical Care**	166 (2.3%)	60 (3.3%)	0.013*	434 (2.5%)	156 (3.6%)	< 0.001***
**PE**	39 (0.5%)	7 (0.4%)	0.416	107 (0.6%)	19 (0.4%)	0.166
**DVT**	52 (0.7%)	11 (0.6%)	0.613	111 (0.6%)	22 (0.5%)	0.317
**Infection**	137 (1.9%)	19 (1.0%)	0.014*	364 (2.1%)	65 (1.5%)	0.011*

^*1*^n (%); ^*2*^Pearson’s Chi-squared test; ^*3*^Fisher’s exact test; *P < 0.05; **P < 0.01; ***P < 0.001

### Logistic regression analysis

Unadjusted propensity score–matched logistic regression demonstrated higher odds of A&E attendance (OR: 1.23; [95% CI: 1.04–1.45]; *P* = 0.013) and hospital readmission (OR: 1.76; [95% CI: 1.39–2.22]; *P* < 0.001) in the day-case group. Regarding complications, the odds of an infection were significantly lower among day-case patients (OR: 0.55; [95% CI: 0.33–0.87]; *P* = 0.015). Similar statistically significant patterns were observed in the unadjusted propensity score–matched logistic regression of the LoS dataset. The full logistic regression results for the patient classification and LoS datasets are shown in Tables 5 and 6, respectively.

**Table 5:** Unadjusted, adjusted, and propensity score–matched logistic regression of 90-day outcomes comparing day-case and inpatient procedures in the Patient Classification dataset. Odds ratios are presented with inpatient procedures as the reference group

	**Unadjusted + Unmatched**			**Adjusted + Unmatched**			**Unadjusted + PSM**		
90-day Outcomes	**OR**	**95% CI**	***P*-value**	**OR**	**95% CI**	***P*-value**	**OR**	**95% CI**	***P*-value**
Any A&E attendance	1.19	1.03, 1.38	**0.016**	1.36	1.17, 1.57	** < 0.001**	1.23	1.04, 1.45	**0.013**
A&E attendance resulting in admission	1.33	1.08, 1.60	**0.005**	1.84	1.50, 2.23	** < 0.001**	1.76	1.39, 2.22	** < 0.001**
Critical Care	1.15	0.88, 1.48	0.279	1.43	1.09, 1.83	**0.008**	1.46	1.07, 1.96	**0.013**
PE	0.59	0.25, 1.14	0.163	0.78	0.34, 1.52	0.522	0.72	0.29, 1.51	0.418
DVT	0.69	0.36, 1.19	0.224	0.93	0.48, 1.60	0.804	0.85	0.42, 1.56	0.613
Infection	0.39	0.24, 0.59	** < 0.001**	0.45	0.28, 0.69	** < 0.001**	0.55	0.33, 0.87	**0.015**

**Table 6:** Unadjusted, adjusted, and propensity score–matched logistic regression of 90-day outcomes comparing day-case (LoS = 0) and inpatient (LoS > 0) procedures in the length of stay (LoS) dataset. Odds ratios are presented with inpatient procedures as the reference group

	**Unadjusted + Unmatched**			**Adjusted + Unmatched**			**Unadjusted + PSM**		
90-day Outcomes	**OR**	**95% CI**	***P*-value**	**OR**	**95% CI**	***P*-value**	**OR**	**95% CI**	***P*-value**
Any A&E attendance	1.20	1.09, 1.31	** < 0.001**	1.29	1.17, 1.41	** < 0.001**	1.21	1.09, 1.34	** < 0.001**
A&E attendance resulting in admission	1.49	1.31, 1.67	** < 0.001**	1.86	1.64, 2.10	** < 0.001**	1.80	1.55, 2.07	** < 0.001**
Critical Care	1.26	1.07, 1.47	**0.005**	1.47	1.24, 1.72	** < 0.001**	1.45	1.20, 1.75	** < 0.001**
PE	0.67	0.41, 1.02	0.081	0.85	0.52, 1.29	0.470	0.71	0.42, 1.13	0.168
DVT	0.58	0.37, 0.86	**0.010**	0.73	0.47, 1.08	0.146	0.79	0.49, 1.23	0.318
Infection	0.56	0.43, 0.71	** < 0.001**	0.62	0.48, 0.79	** < 0.001**	0.71	0.54, 0.92	**0.012**

### Long-term survival analysis

Univariate Cox hazards regression demonstrated significantly greater survival probabilities for patients coded as day-case arthroplasty procedures compared to inpatient procedures (HR: 0.59; [95% CI: 0.50–0.69]; *P* < 0.001). This significant association persisted in multivariate regression analysis, albeit to a lower extent (HR: 0.84; [95% CI: 0.72–0.99]; *P* = 0.034).

Similar findings were noted in the LoS dataset, with both univariate (HR: 0.68; [95% CI: 0.62–0.75]; *P* < 0.001) and multivariate Cox regression analysis highlighting reduced hazard of post-operative mortality in patients undergoing day-case arthroplasty (HR: 0.86; [95% CI: 0.78–0.95]; *P* = 0.002) (Table 7).

**Table 7:** Univariate and multivariate Cox hazard regression models of survival comparing day-case and inpatient procedures

	**Univariate Model**			**Multivariate Model**		
	**HR**	**95% CI**	***P*-value**	**HR**	**95% CI**	***P*-value**
**Patient Classification**						
Inpatient	[Ref]			[Ref]		
Day-case	0.59	0.50, 0.69	** < 0.001**	0.84	0.72, 0.99	**0.034**
**Length of Stay (days)**						
> 0	[Ref]			[Ref]		
=0	0.68	0.62, 0.75	** < 0.001**	0.86	0.78, 0.95	**0.002**

CI = Confidence Interval, HR = Univariate Cox Proportional Hazard Ratio

### Kaplan–Meier survival curves

**Fig. 3:**
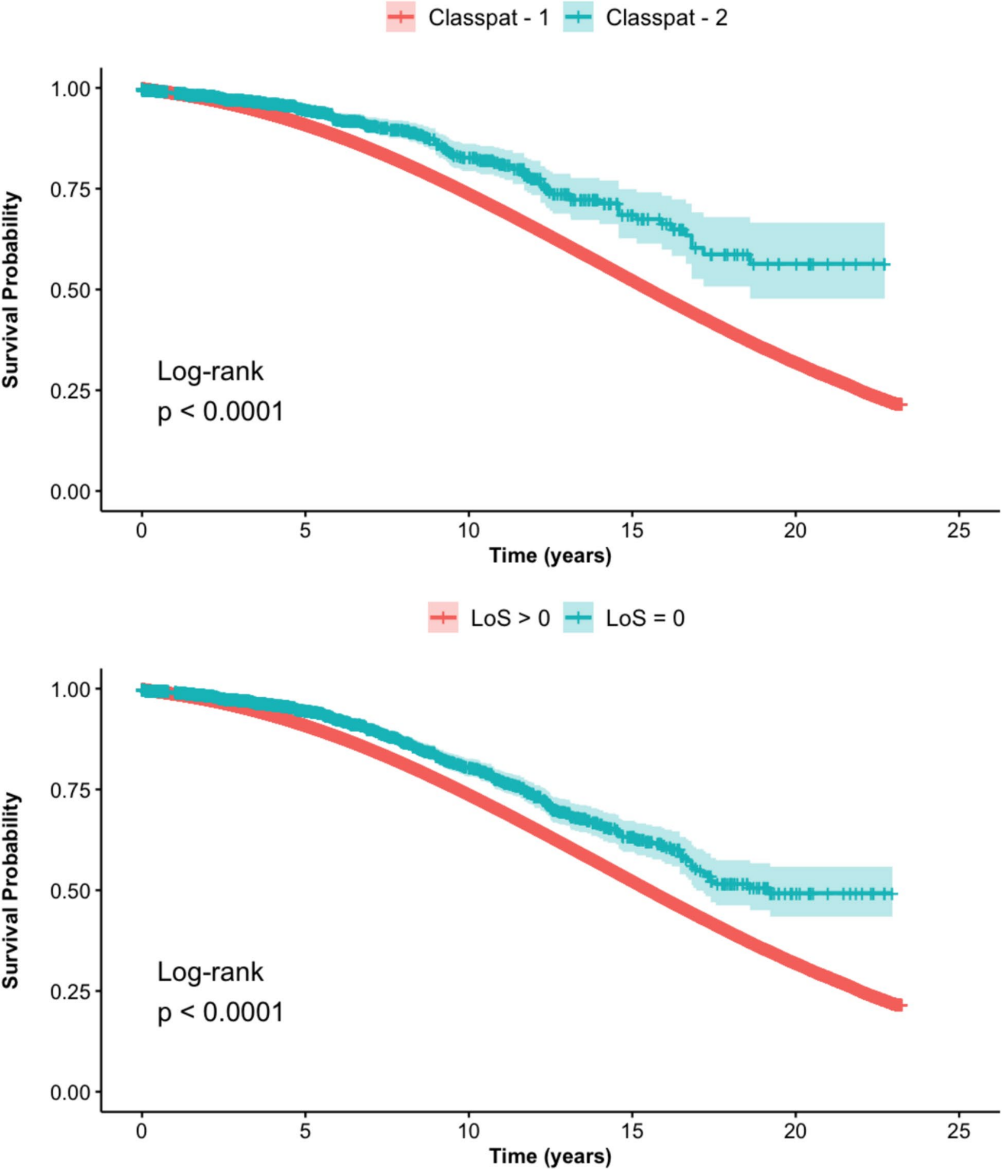
**(A)** Kaplan–Meier survival curves comparing day-case and inpatient procedures in the Patient Classification dataset; (**B**) Kaplan–Meier survival curves comparing day-case and inpatient procedures in the length of stay (LoS) dataset

As seen in the Kaplan–Meier survival curves for the patient classification and LoS datasets (Figs. 3A and B), respectively, day-case arthroplasty is associated with significantly higher long-term survival probabilities compared to the inpatient pathway (*P* < 0.001).

### Consultant level analysis

Of the 6,563 consultant codes tagged to the hip and knee arthroplasties, 5,410 (82.4%) were tagged as T&O (specialty code = 110) and had non-null consultant codes (non- ‘&’ or ‘99’). Of these, only 481 (8.9%) performed at least one day-case pathway operation.

Of the 481, the proportion of operations carried out as planned day cases ranged from 0.02% to 100%. While all day-cases, including zero LoS, ranged from 0.03% to 100%, with 992 consultants performing them.

When examining the number of day-case versus non-day-case by geographic region, statistically significant differences were observed (χ^2^ = 672.71, df = 8, *P* < 0.001). Yorkshire and Humber had the highest proportion, at 0.64%, and the West Midlands had the lowest, at 0.22%. Post-hoc inspection of standardised residuals (stdres) identified the regions contributing most to this association. Patients classified as day-case were significantly over-represented in the East Midlands (stdres = 10.88, *P* < 0.001), South East (4.83, *P* < 0.001), and particularly in Yorkshire and the Humber (15.26, *P* < 0.001). In contrast, day-case surgery was markedly under-represented in the East of England (− 4.48, *P* < 0.001), London (− 2.53, *P* < 0.05), North West (− 2.65, *P* < 0.05), and most notably in the West Midlands (− 9.47, *P* < 0.001).

## Discussion

To the best of our knowledge, this is the first national-level study that directly examines the safety and clinical efficacy of day-case hip and knee arthroplasty within the context of the NHS. With a sample size of 1,148,293 procedures, we leveraged the largest linked arthroplasty dataset in the United Kingdom, providing a comprehensive overview of the feasibility of day-case arthroplasty. Our study highlights several crucial findings, most notably that, to date, day-case arthroplasty in the UK is safe and feasible but associated with significantly higher A&E attendances and readmission rates, as well as admissions to critical care, within the initial 90-days post-operatively. This is set against lower rates of deep vein thrombosis and infection in the day-case patients. Additionally, long-term survival was found to be significantly longer in patients undergoing day-case arthroplasty. This finding may be attributed to selection bias, with day-case patients being younger and healthier, with fewer comorbidities. However, the survival benefit was sustained after matching for key demographic variables, including age, gender, ethnicity, and comorbidities.

The existing UK literature surrounding the safety and efficacy of day-case hip and knee arthroplasty presents mixed findings. In a retrospective cohort study of 46 NHS patients, Gao et al. found that those undergoing day-case UKA had significantly lower reattendance rates and fewer complications compared to patients managed as inpatients, a finding that is inconsistent with our results [15]. One possible explanation for this discrepancy is that their small cohort was limited to UKA patients, compared to our inclusion of THA, TKA, UKA, and HRA procedures. Considering the less invasive nature of UKA, with lower operative times, intraoperative blood loss, length of stay, and favourable safety profile compared to TKA, it is unsurprising that studies focusing exclusively on UKA in the day-case setting have reported better outcomes [20]. Indeed, in French et al.’s recent meta-analysis, UKA was the only procedure to show a statistical advantage over inpatient surgery, albeit this was only in the domain of reduced post-operative complications [21].

Furthermore, the demographics of Gao et al.’s two cohorts were notably different, with day-case patients being substantially younger, with a lower BMI and ASA status, which may also have contributed to the superior outcomes observed in the day-case cohort [15]. The lack of adjustment for potential confounding variables limits the validity of their results and simultaneously highlights a major strength of our study, with the incorporation of propensity score matching. Another UK-based cohort study by Fishley et al. reported that, among 301 patients undergoing day-case TKA, 28 patients (9.3%) attended A&E and 6 patients (2.0%) were readmitted, which is comparable to our findings [16]. However, this study was limited to three hospitals within the same geographical region, whereas ours encompassed the entire United Kingdom.

While evidence on day-case hip and knee arthroplasty in the United Kingdom is limited, a substantially larger body of literature exists in North America. The systematic review by French et al., consisting of 38 studies predominantly from the USA and Canada, reported that day-case procedures were associated with lower odds of A&E admission (OR: 0.62; [95% CI 0.48–0.79]; *P* < 0.001), emergency readmission (OR: 0.83; [95% CI: 0.73–0.96]; *P* = 0.009), and complications (OR: 0.7; [95% CI: 0.55–0.89]; *P* = 0.004) compared to the inpatient pathway [21]. The contrast with our findings is clear and suggests that a granular comparison of techniques and approaches is needed to understand why these differences exist. Countries such as the US and Denmark have been at the forefront of developing day-surgery protocols in terms of patient selection, peri-operative care, and post-operative monitoring. It may be that these lessons have not been transferred to the UK, particularly during the earlier part of the period we studied, which arguably covers the start of the day surgery arthroplasty revolution in the UK, as seen in the percentage of day-cases over time (Fig. 1), and also in a recent National Joint Registry-based analysis [22]. Additionally, only 41.8% of patients identified by a LoS of zero days were also coded as “day surgery”, suggesting that these patients may have been opportunistic discharges on the day of surgery, and did not follow a predetermined day surgery pathway with appropriate pre-operative patient information, peri-operative anaesthetic and surgical techniques, and post-discharge support systems. The argument against this, explaining our unexpected findings of higher readmission/A&E/critical care events in day surgery patients, is that our findings were almost identical in the cohort who were planned and coded as ‘day surgery’ patients. Regarding our dataset, analysing the optimal LOS required to achieve favourable post-operative outcomes would have provided valuable insight and represents an important area for future research. Moreover, data on patients who experienced successful versus failed day-case procedures would provide important contextual insight. In particular, identifying the specific variables associated with day-case failure, as well as factors linked to increased readmission rates and A&E attendance, is crucial for optimising patient selection and improving clinical outcomes. Unfortunately, our dataset also lacked longitudinal data on how outcomes from day-case procedures have evolved, which would have provided valuable insights into lessons learned and ongoing challenges that could inform future practice.

Another possible factor contributing towards disparities in admission outcomes, particularly within the US literature, is variation in the definition of day-case arthroplasty. In this study, we defined day-case arthroplasty as discharge on the day of surgery. However, several other studies have used alternative definitions, with inpatient stays lasting up to 24 h [23]. Such definitional differences have been shown to affect study outcomes and may explain the disparities between our findings and those reported in the literature [23]. However, French et al.’s meta-analysis only included studies with discharge on the day of surgery, which suggests that this variable does not explain our differing findings.

The consultant-level analysis revealed that the overall association between region and day-case status is driven by pronounced geographic disparities, with certain regions showing strong deviations from expected distributions under the null hypothesis. This is consistent with a UK-based retrospective analysis by French et al., which highlighted that a small number of units accounted for the majority of day-case procedures, with the five highest volume centres alone performing 48.6% of such cases in 2021 [21]. In this context, it remains difficult to determine whether these disparities are due to underlying regional factors such as socioeconomic or demographic variation. As such, given the currently low proportion of day surgery procedures, differences in departmental goals, infrastructure, and resources will contribute to the observed disparities in volume across the nation.

While this study provides novel insights, several limitations should be acknowledged. First, the definition of day-case success was confined to survival without A&E attendance, readmission, critical care admission, or death within 90 days. Although these are clear, measurable, and important events, this definition does not include the broader aspects of recovery, such as functional improvement or patient satisfaction. The lack of information on medication use, along with possible variations in physiotherapy and rehabilitation participation, may also have influenced the observed complication rates. Moreover, within the group of patients with a LoS of zero days, there is a possibility that some may have self-discharged for unrelated reasons, representing another limitation of the study. Lastly, despite our large study cohort, only a small percentage of these cases were day-case procedures, and this ultimately hinders the validity of our findings.

## Conclusion

In conclusion, this national cohort study demonstrates that day-case hip and knee arthroplasty in the NHS carries a higher risk of hospital readmission and critical care admittance compared to the traditional inpatient pathway. It is also associated with an increased chance of A&E attendance and readmission. However, it is associated with reduced deep vein thrombosis and infection rates and improved long-term survival outcomes, even after matching for key demographic variables. Although expansion of day-case pathways in the UK is appealing to address capacity constraints and reduce elective care backlogs, implementation must be accompanied by careful patient selection, robust perioperative protocols, and strategies to mitigate downstream emergency service burden. Future research should focus on defining best practices, such as refining selection criteria, optimising day surgery techniques, post-discharge support, and assessing patient-reported outcomes, to ensure that the benefits of day-case arthroplasty are realised without compromising safety and quality of care. Additionally, examining the cost-effectiveness of day-case arthroplasty represents an important area for future research to assess its suitability for integration within the NHS, particularly in the context of ongoing resource limitations.

## Supplementary Information

Supplementary Material 1. Table S1. ICD-10 Codes Used to Identify Complications.

## Data Availability

No datasets were generated or analysed during the current study.

## References

[1] National Joint Registry Annual Reports. National Joint Registry © National Joint Registry 2024. 2023. Available from: https://reports.njrcentre.org.uk/2023.

[2] Matharu GS, Culliford DJ, Blom AW, Judge A. Projections for primary hip and knee replacement surgery up to the year 2060: an analysis based on data from The National Joint Registry for England, Wales, Northern Ireland and the Isle of Man. Ann R Coll Surg Engl. 2022;104(6):443–8. 10.1308/rcsann.2021.0206. 34939832 10.1308/rcsann.2021.0206 PMC9157920

[3] Wainwright TW, Immins T, Middleton RG. Changes in hip and knee arthroplasty practice post-COVID-19 in the English NHS: a retrospective analysis of hospital episode statistics data. Ann R Coll Surg Engl. 2025;107(4):253–6. 10.1308/rcsann.2024.0100. 39570318 10.1308/rcsann.2024.0100 PMC11957841

[4] Jabbal M, Burt J, Clarke J, Moran M, Walmsley P, Jenkins PJ. Trends in incidence and average waiting time for arthroplasty from 1998-2021: an observational study of 282,367 patients from the Scottish arthroplasty project. Ann R Coll Surg Engl. 2024;106(3):249–55. 10.1308/rcsann.2023.0039. 37365920 10.1308/rcsann.2023.0039 PMC10911452

[5] Desmeules F, Dionne CE, Belzile E, Bourbonnais R, Frémont P. The burden of wait for knee replacement surgery: effects on pain, function and health-related quality of life at the time of surgery. Rheumatology (Oxford). 2010;49(5):945–54. 10.1093/rheumatology/kep469. 20144931 10.1093/rheumatology/kep469

[6] Reforming elective care for patients 2025. Available from: https://www.england.nhs.uk/publication/reforming-elective-care-for-patients/.

[7] Thompson JW, Wignadasan W, Ibrahim M, Beasley L, Konan S, Plastow R, et al. Day-case total hip arthroplasty: a literature review and development of a hospital pathway. Bone & Joint Open. 2021;2(2):93–102. 10.1302/2633-1462.22.Bjo-2020-0170.R1. 33573396 10.1302/2633-1462.22.BJO-2020-0170.R1 PMC7925215

[8] NHS Hospital Beds Data Analysis (2022) British Medical Association. Available at: https://www.bma.org.uk/advice-and-support/nhs-delivery-and-workforce/pressures/nhs-hospital-beds-data-analysis.

[9] Richardson MK, Wier J, Liu KC, Mayfield CK, Vega AN, Lieberman JR, et al. Same-day total joint arthroplasty in the United States from 2016 to 2020: the impact of the Medicare Inpatient Only List and the COVID-19 pandemic. J Arthroplasty. 2024;39(4):858-63.e2. 10.1016/j.arth.2023.10.025. 37871863 10.1016/j.arth.2023.10.025

[10] French JMR, Deere K, Sayers A, Whitehouse MR. Day case hip and knee replacement in England: a population-based cohort study using linked National Joint Registry and Hospital Episode Statistics data. BMC Med. 2025;23(1):564. 10.1186/s12916-025-04280-y. 41088192 10.1186/s12916-025-04280-y PMC12523212

[11] Dey S, Gadde R, Sobti A, Macdonald N, Jacob J, Unnithan A. The safety and efficacy of day-case total joint arthroplasty. Ann R Coll Surg Engl. 2021;103(9):638–44. 10.1308/rcsann.2021.0066. 33851548 10.1308/rcsann.2021.0066 PMC10335200

[12] Hlatshwako TG, Jenkins C, Wordsworth S, Murray D, Barker K, Dakin H. Using orthopaedic health care resources efficiently: a cost analysis of day surgery for unicompartmental knee replacement. Knee. 2024;49:147–57. 10.1016/j.knee.2024.06.006. 38964260 10.1016/j.knee.2024.06.006 PMC7616244

[13] Patel KT, Lewis TL, Gill P, Chatterton M. The patient perspective, experience and satisfaction of day case unicompartmental knee arthroplasty: a short-term mixed-methods study. Knee. 2021;33:378–85. 10.1016/j.knee.2021.10.022. 34775281 10.1016/j.knee.2021.10.022

[14] Courtney PM, Boniello AJ, Berger RA. Complications following outpatient total joint arthroplasty: an analysis of a national database. J Arthroplasty. 2017;32(5):1426–30. 10.1016/j.arth.2016.11.055. 28034481 10.1016/j.arth.2016.11.055

[15] Gao N-P, Al-Dadah O. Comparison of day-case versus inpatient uni-compartmental knee replacement. Musculoskelet Care. 2023;21(1):16–24. 10.1002/msc.1662. 10.1002/msc.1662 35652292

[16] Fishley WG, Paice S, Iqbal H, Mowat S, Kalson NS, Reed M, et al. Low readmission and reattendance rate in day-case total knee arthroplasties. Bone & Joint Open. 2023;4(8):621–7. 10.1302/2633-1462.48.BJO-2023-0043.R1. 37604493 10.1302/2633-1462.48.BJO-2023-0043.R1 PMC10442176

[17] Saunders P, Smith N, Syed F, Selvaraj T, Waite J, Young S. Introducing a day-case arthroplasty pathway significantly reduces overall length of stay. Bone & Joint Open. 2021;2(11):900–8. 10.1302/2633-1462.211.Bjo-2021-0106.R1. 34729998 10.1302/2633-1462.211.BJO-2021-0106.R1 PMC8636294

[18] Bottle A, Aylin P. Comorbidity scores for administrative data benefited from adaptation to local coding and diagnostic practices. J Clin Epidemiol. 2011;64(12):1426–33. 10.1016/j.jclinepi.2011.04.004. 21764557 10.1016/j.jclinepi.2011.04.004

[19] van Walraven C, Austin PC, Jennings A, Quan H, Forster AJ. A modification of the Elixhauser comorbidity measures into a point system for hospital death using administrative data. Med Care. 2009;47(6):626–33. 10.1097/MLR.0b013e31819432e5. 19433995 10.1097/MLR.0b013e31819432e5

[20] Suarez JC, Saxena A, Arguelles W, Watson Perez JM, Ramamoorthy V, Hernandez Y, et al. Unicompartmental knee arthroplasty vs total knee arthroplasty: a risk-adjusted comparison of 30-day outcomes using national data from 2014 to 2018. Arthroplasty Today. 2022;17:114–9. 10.1016/j.artd.2022.06.017. 36082284 10.1016/j.artd.2022.06.017 PMC9445223

[21] French JMR, Woods A, Sayers A, Deere K, Whitehouse MR. Day-case knee and hip replacement. The Bone & Joint Journal. 2024;106-B(12):1385–92. 10.1302/0301-620X.106B12.BJJ-2024-0021.R1. 39615519 10.1302/0301-620X.106B12.BJJ-2024-0021.R1

[22] Hampton M, Clark M, Baxter I, Stevens R, Flatt E, Murray J, et al. The effects of a UK lockdown on orthopaedic trauma admissions and surgical cases: a multicentre comparative study. Bone Jt Open. 2020;1(5):137–43. 10.1302/2633-1462.15.Bjo-2020-0028.R1. 33241224 10.1302/2633-1462.15.BJO-2020-0028.R1 PMC7684391

[23] Bovonratwet P, Webb ML, Ondeck NT, Lukasiewicz AM, Cui JJ, McLynn RP, et al. Definitional differences of ‘outpatient’ versus ‘inpatient’ THA and TKA can affect study outcomes. Clin Orthop Relat Res. 2017;475(12):2917–25. 10.1007/s11999-017-5236-6. 28083753 10.1007/s11999-017-5236-6 PMC5670045

