# Supplementary material for: Safety and efficacy of day-case hip and knee arthroplasty in the NHS: a nationwide UK cohort study
Source: Arthroplasty. 2026 Mar 13;8:20. doi: 10.1186/s42836-026-00377-8 (PMC12983480; doi:10.1186/s42836-026-00377-8)
Supplement: Supplementary file 1 — Supplementary Material 1. Table S1. ICD-10 Codes Used to Identify Complications. [file 42836_2026_377_MOESM1_ESM.docx]

**Supplementary Tables**:

**Table S1.** ICD-10 Codes Used to Identify Complications

| **Domain** | **ICD-10 Codes** |
| --- | --- |
| PE | I26* |
| DVT | I80.1-I80.4, I80.8, I80.9, I82.1, I82.8, I82.9 |
| Infection | T81.4, T81.5, T81.6, T82.7, T85.7, T88.7, L03.1, T84.5, T84.7, A40, A41 |

PE = Pulmonary Embolism; DVT = Deep Vein Thrombosis. *All codes with the three digit alphanumeric codes, encompassing all codes after the decimal point.
